# RelQ-mediated alarmone signalling regulates growth, stress-induced biofilm formation and spore accumulation in Clostridioides difficile

**DOI:** 10.1099/mic.0.001479

**Published:** 2024-07-19

**Authors:** Areej Malik, Adenrele Oludiran, Asia Poudel, Orlando Berumen Alvarez, Charles Woodward, Erin B. Purcell

**Affiliations:** 1 Biomedical Sciences Program, Old Dominion University, Norfolk, Virginia, 23529, USA; 2 Department of Chemistry and Biochemistry, Old Dominion University, Norfolk, Virginia, 23529, USA

**Keywords:** alarmone, biofilm, *Clostridioides difficile*, stringent response

## Abstract

The bacterial stringent response (SR) is a conserved transcriptional reprogramming pathway mediated by the nucleotide signalling alarmones, (pp)pGpp. The SR has been implicated in antibiotic survival in *Clostridioides difficile*, a biofilm- and spore-forming pathogen that causes resilient, highly recurrent *C. difficile* infections. The role of the SR in other processes and the effectors by which it regulates *C. difficile* physiology are unknown. *C. difficile* RelQ is a clostridial alarmone synthetase. Deletion of *relQ* dysregulates *C. difficile* growth in unstressed conditions, affects susceptibility to antibiotic and oxidative stressors and drastically reduces biofilm formation. While wild-type *C. difficile* displays increased biofilm formation in the presence of sublethal stress, the Δ*relQ* strain cannot upregulate biofilm production in response to stress. Deletion of *relQ* slows spore accumulation in planktonic cultures but accelerates it in biofilms. This work establishes biofilm formation and spore accumulation as alarmone-mediated processes in *C. difficile* and reveals the importance of RelQ in stress-induced biofilm regulation.

## Introduction

*Clostridioides difficile*is a Gram-positive, biofilm- and spore-forming obligate anaerobic pathogen that causes *C. difficile* infection (CDI) [1]. This pathogen colonizes the mammalian large intestine after the disruption of beneficial commensal bacteria due to antibiotic treatment or advanced age [2]. Antibiotic treatment for CDI further exacerbates the dysbiosis of the intestinal microbiota, resulting in the recurrence of CDI [3]. The chance of recurrent infection increases from 30 % to 45 % to 64 % after each successive antibiotic treatment for CDI, a phenomenon known as the ‘recurrence elevator’ [3]. In the USA, *C. difficile* is responsible for approximately 500 000 infections, 25 000 deaths and roughly $6 billion in hospitalization costs every year [4]. Patients with severe or recurring CDIs are commonly treated with the antibiotics fidaxomicin, vancomycin or metronidazole [5].

The bacterial stringent response (SR) is a conserved transcriptional regulatory mechanism activated when bacteria are exposed to stressful conditions. It allows bacteria to rapidly adjust to stress by temporarily inhibiting growth and cell division while simultaneously prompting the transcription of genes involved in stress survival [6,7]. In diverse microbes, the bacterial SR may be involved in tolerance of antibiotics, survival against antimicrobial peptides, inhibiting growth, resistance to oxidative stress, expression of genes governing virulence traits such as biofilm formation and sporulation and/or competitive advantage against other microbes [8].

The SR is controlled by the accumulation of intracellular alarmone nucleotides that are collectively known as (pp)pGpp [9,10]. These alarmones are metabolized by bifunctional synthetase/hydrolase enzymes from the RSH or Rel (RelA/SpoT homology) family, monofunctional small alarmone hydrolases (SAHs), and monofunctional small alarmone synthetases (SASs) [11]. The SR is induced when bacteria are exposed to extracellular stresses that can include but are not limited to nutrient deprivation, antibiotic treatment or oxidative stress [6,12, 13]. Firmicutes species generally encode only one RSH/Rel but can have one or two SASs, typically from the RelP and RelQ families [11]. The highly conserved synthetase domains of RSH/Rel and SAS enzymes transfer a pyrophosphate from ATP to the 3′ OH of guanosine nucleotide precursors. Typically, the utilization of guanosine-5’- monophosphate (GMP), guanosine-5’- diphosphate (GDP) or guanosine-5’- triphosphate (GTP) results in the synthesis of pGpp, ppGpp or pppGpp, respectively [9,10]. *C. difficile* encodes highly conserved RSH/Rel and RelQ homologs whose alarmone synthesis contributes to antibiotic survival [10,14, 15]. However, *in vitro* studies with purified proteins recently revealed that both C. *difficile* synthetases must hydrolyse a 5′ phosphate or pyrophosphate from their GDP or GTP substrates while transferring the ATP-derived 3′ pyrophosphate, leaving behind a 5′ monophosphate and resulting in exclusive synthesis of pGpp [10,14, 15].

Biofilm formation can be triggered by extracellular stresses. The process starts off with planktonic (free-floating) cells setting and attaching onto solid surfaces in single-species or polymicrobial communities [16]. Biofilms are multilayered and have a protective matrix surrounding the cells, which is made up of bacterial proteins, extracellular DNA and polysaccharides and protects the cells from antibiotics and host immune responses [17,18]. Compared to free-living cells, cells within biofilms also undergo physiological changes such as decreased growth rate and altered transcriptional activity [19]. The ability of *C. difficile* to form biofilms is one of the reasons that the pathogen can survive antibiotic treatment or exposure to immune stresses *in vivo* [17,20, 21]. The structure and composition of biofilm are dependent on the strain and biofilm age [17,18]. Some *C. difficile* strains are able to form more robust biofilms *in vivo*, resulting in higher antibiotic resistance [22]. *C. difficile* strains isolated from patients with recurring CDIs produce more biofilms than strains isolated from patients whose CDIs resolved after one round of antibiotic treatment [23]. This suggests that *C. difficile* biofilms serve as a reservoir for recurrent infections [23,24]. It has also been shown that sublethal concentrations of the antibiotics vancomycin and metronidazole stimulate biofilm formation, thereby reducing antibiotic susceptibility and increasing the persistence and recurrence of CDIs [17,25, 26].

The ability to form spores is critical for the dissemination of *C. difficile* into the surrounding environment and spread to new patients [27,28]. The protective bacterial spore structure has characteristics that are different from those of a vegetative cell, which aid in bacterial resistance and durability in the environment [29,30]. Many of the layers surrounding the spore core contribute to bacterial spore resistance [28,30,35]. The spore core is highly dehydrated and composed of DNA, enzymes, tRNA and ribosomes [30]. It is known that antibiotics do not eradicate spores [36].

While metabolically active vegetative *C. difficile* cells are obligate anaerobes and are killed within minutes by exposure to atmospheric oxygen, spores can survive desiccation and exposure to environmental oxygen for months [37]. Within the intestinal tract of a susceptible host, *C. difficile* spores germinate after exposure to primary bile acids and the amino acids alanine and glycine [38]. Beneficial commensal microbes create an environment inhospitable to *C. difficile* colonization by depleting nutrients such as amino acids and converting primary bile acids into secondary bile acids, but these protective factors are diminished when advanced age or antibiotic treatment disrupts the intestinal microbiota [39,40].

While biofilm formation and sporulation are divergent developmental fates for an individual cell, they are complementary processes for a population. The majority of cells in 3-day-old *C. difficile* biofilms are vegetative cells, but spores outnumber vegetative cells in 6-day-old biofilms [18,21]. These results indicate that biofilms can serve as reservoirs for spores, thus increasing bacterial resistance to a variety of treatments [24,41]. While both processes are important for disease persistence, recurrence and transmission, neither is completely understood in *C. difficile*. In spite of the high clinical significance of *C. difficile* infection, the signalling pathway that results in the transition of a dormant spore to a metabolically active vegetative cell is not completely understood in *C. difficile* [42]. Some regulators that affect sporulation have been identified but lack conservation with other sporulating bacteria, and it is still not known how these regulators respond to external conditions [43]. Similarly, it is known that biofilm formation is affected by the nucleotide signals c-di-GMP and c-di-AMP in *C. difficile*, but the extracellular signals that trigger their synthesis and degradation are mostly unknown [44,45].

The SR is conserved across almost all bacterial species, but the specific activating signals, effectors and the resulting phenotypes governed by the SR vary between species [8,46]. The SR has been linked to virulence and biofilm formation in multiple bacterial species, including *Streptococcus mutans*, *Pseudomonas aeruginosa*, *Enterococcus faecalis*, *Vibrio cholerae*, *Bordetella pertussis* and *Escherichia coli* [47,53]. The SR also plays a significant role in the tolerance of *P. aeruginosa* and *Staphylococcus aureus* biofilms to antibiotics and may stimulate biofilm formation in *S. aureus* [48,54,57]. The SR also affects sporulation in *Bacillus subtilis* and antibiotic tolerance in *C. difficile* [15,58]. Antibiotic exposure, oxidative stress and metal starvation have been shown to upregulate transcription of clostridial synthetase genes, with *rsh* being more transcriptionally responsive than *relQ* [14,15]. Here, we deleted the *relQ* gene in *C. difficile* 630Δ*erm* and observed that the deletion strain has reduced biofilm formation in all the conditions that were studied and has lost the ability to upregulate biofilm formation in response to stress. We further report that *relQ* deletion delays spore accumulation in biofilm supernatants and stimulates spore accumulation in adherent biofilms. While RelQ is not the only clostridial alarmone synthetase, it does have a readily discernible role in regulating stress-responsive biofilm formation and spore accumulation in *C. difficile*.

## Results

### *relQ* regulates *C. difficile* 630Δ*erm* growth

**Fig. 1.**
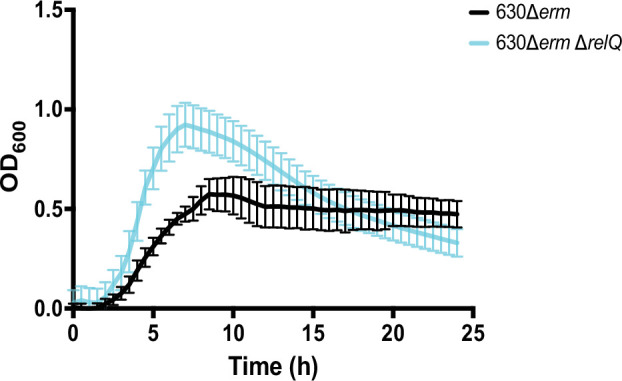
*C. difficile* 630Δ*erm* and 630Δ*erm*Δ*relQ* growth in BHIS broth. Growth in a 96-well plate of wild-type and deletion strain cultures in BHIS broth over 24 h. Data represent the mean and standard deviation of at least eight independent biologicals measured as duplicates.

Allele-coupled exchange (ACE) was used to excise the *relQ* open reading frame from the *C. difficile* 630Δ*erm* genome (Fig. S1, available in the online version of this article). Growth of *C. difficile* 630Δ*erm* (wild-type) and 630Δ*erm* Δ*relQ* (deletion) strains was compared in brain heart infusion supplemented (BHIS) broth. The duration of the lag phase, before exponential growth becomes visible, is similar for wild-type and deletion strains (Fig. 1). Once growth begins, the strains rapidly diverge. The wild-type strain exhibits ~4 h of exponential growth with a doubling time of 1.5 h, followed by a transition into stationary phase at an optical density at 600 nm (OD_600_) of 0.55. The OD_600_ remains stable at 0.5 for the remainder of the 24 h experiment (Fig. 1). By contrast, the deletion strain has a doubling time of 0.79 h, until it reaches an OD_600_ of 0.95, followed by a continuous decline through the rest of the 24 h experiment (Fig. 1). Within 16 h, the deletion strain cell density has dropped below that of the wild-type strain (Fig. 1).

### *relQ* deletion has non-uniform effects on *C. difficile* 630Δ*erm* growth during stress

We have previously demonstrated that the activity of the *relQ* promoter in *C. difficile* strains 630Δ*erm* and R20291 is stimulated by 2 h of exposure to sub-inhibitory concentrations of antibiotics in a strain-specific manner [14,15]. The *relQ* promoter was responsive to fidaxomicin in R20291 and to vancomycin in 630Δ*erm* but did not respond to metronidazole in either strain [14,15]. To assess the impact of *relQ* on the cellular response to non-lethal antibiotic stress, we compared the growth of the wild-type and deletion strains in the presence of antibiotics.

**Fig. 2.**
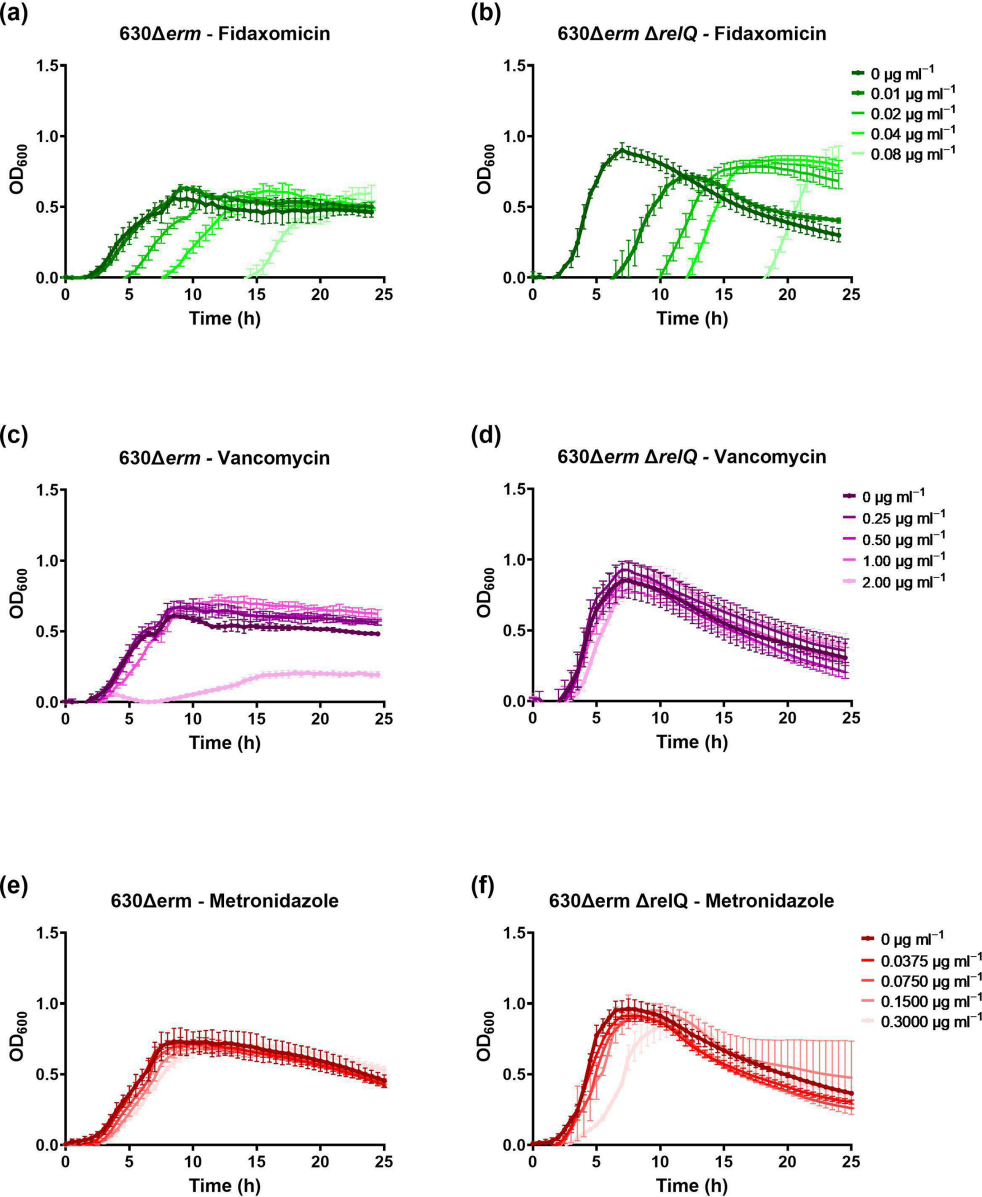
Effect of sublethal antibiotics on growth of the *C. difficile* 630Δ*erm* and 630Δ*erm* Δ*relQ* strains. Growth of wild-type and deletion strain cultures in the presence of indicated concentrations of (**a and b**) fidaxomicin, (**c and d**) vancomycin and (**e and f**) metronidazole. Data represent the mean and standard deviation of three independent biologicals measured as duplicates.

We found that while the wild-type and deletion strains exhibit different growth kinetics, both are delayed for several hours by the initial presence of a single dose of fidaxomicin (Fig. 2a, b). The deletion strain is slightly more sensitive to the antibiotic, exhibiting growth delays at concentrations of 0.01 µg ml^−1^ and above, while the wild-type strain shows no growth delay below 0.02 µg ml^−1^ (Fig. 2a, b). Similarly, 0.08 µg ml^−1^ fidaxomicin, the highest concentration tested, delayed wild-type growth by 14 h and deletion growth by 18 h (Fig. 2a, b). Both the wild-type and deletion strains showed a negligible response to 0.25, 0.5 and 1.0 µg ml^−1^ vancomycin concentrations (Fig. 2c, d). The highest tested vancomycin concentration, 2.0 µg ml^−1^, meaningfully suppressed wild-type growth for 24 h but had no apparent effect on deletion growth (Fig. 2c, d). The wild-type strain showed no negligible response to all the metronidazole concentrations that were tested (Fig. 2e). Unlike the wild-type strain, the deletion strain was slightly more sensitive to metronidazole, showing a modest growth defect at the concentration of 0.30 µg ml^−1^ (Fig. 2f).

**Fig. 3.**
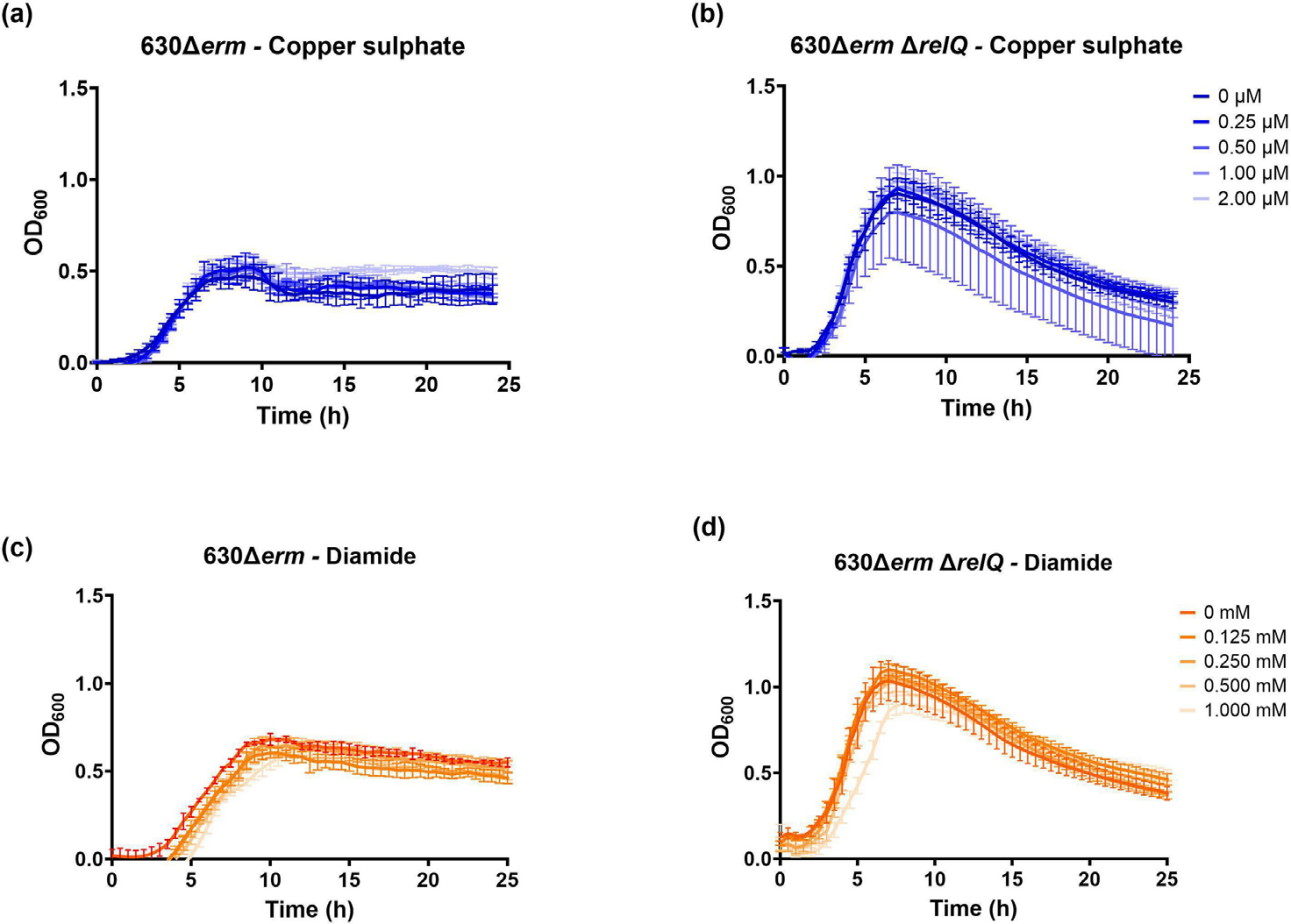
Effect of sublethal oxidants on the growth of *C. difficile* 630Δ*erm* and 630Δ*erm*Δ*relQ* strains. Growth of wild-type and deletion strain cultures in the presence of indicated concentrations of (**a and b**) copper sulphate and (**c and d**) diamide. Data represent the mean and standard deviation of three independent biologicals measured as duplicates.

As an anaerobe, *C. difficile* is vulnerable to oxidative stress from molecular oxygen and transition metals [59,60]. Copper sulphate, a transition metal, can be used to create oxidative stress in an anaerobic environment [60]. In the past, our laboratory has demonstrated that 4.0 µM copper sulphate inhibits *C. difficile* 630Δ*erm* growth, although its effect on *rsh* and *relQ* transcription has not been assessed [60]. Diamide simulates exposure to oxidative stress by initiating the formation of disulphide bonds in proteins [61]. We have previously shown that 3 h treatment with 1.0 mM diamide activated the clostridial *rsh* promoter, although other stressors such as copper sulphate did not affect the transcription of clostridial synthetases [14]. Here, we tested the effect of sub-inhibitory exposure to copper sulphate and diamide on the growth of the wild-type and deletion strains. Neither oxidant affected wild-type growth (Fig. 3a, c). A slight decrease in growth of the deletion strain was observed when treated with 0.50 µM copper sulphate or 1.0 mM diamide (Fig. 3b, d). The absence of *relQ* disrupts the regulation of *C. difficile* 630Δ*erm* growth under normal conditions. Nevertheless, the impact of *relQ* deletion on cellular responses to sublethal stresses examined in this study was varied. In the absence of *relQ*, *C. difficile* 630Δ*erm* displays increased susceptibility to fidaxomicin and less susceptibility to vancomycin. However, *relQ* deletion does not meaningfully affect the growth of *C. difficile* 630Δ*erm* in the presence of metronidazole or oxidative stress.

### *relQ* regulates *C. difficile* 630Δ*erm* stress-responsive biofilm formation

**Fig. 4.**
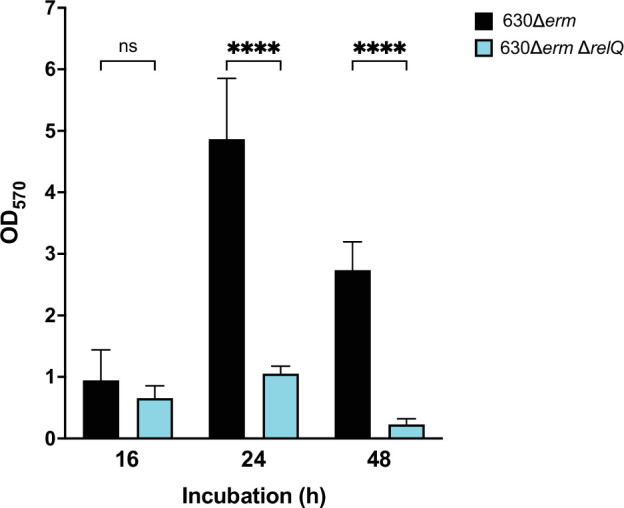
Biofilm production by *C. difficile* 630Δ*erm* and 630Δ*erm*Δ*relQ* strains. Biofilms were grown for 16, 24 or 48 h before washing and staining with crystal violet. Data represent the mean and standard deviation of at least four independent biologicals measured in duplicate. Wild-type and deletion measurements at each timepoint were compared by one-way ANOVA. ****P<0.0001; ns, not significant.

To determine whether RelQ-mediated alarmone signalling affects biofilm production in *C. difficile* 630*Δerm*, biofilms were grown in BHIS broth for 16, 24 and 48 h. After 16 h, no significant difference in biofilm formation was observed between the strains (Fig. 4). The wild-type strain showed substantially increased biofilm formation at 24 h, but the deletion strain did not (Fig. 4). Biofilm formation by both strains decreased between 24 and 48 h but remained much higher for the wild-type strain (Fig. 4).

It has previously been shown that sublethal concentrations of antibiotics, including metronidazole and vancomycin, stimulate *C. difficile* biofilm formation [17,25, 26]. Our next step was to determine if *relQ* deletion affected the upregulation of biofilm formation as a response to specific stresses.

**Fig. 5.**
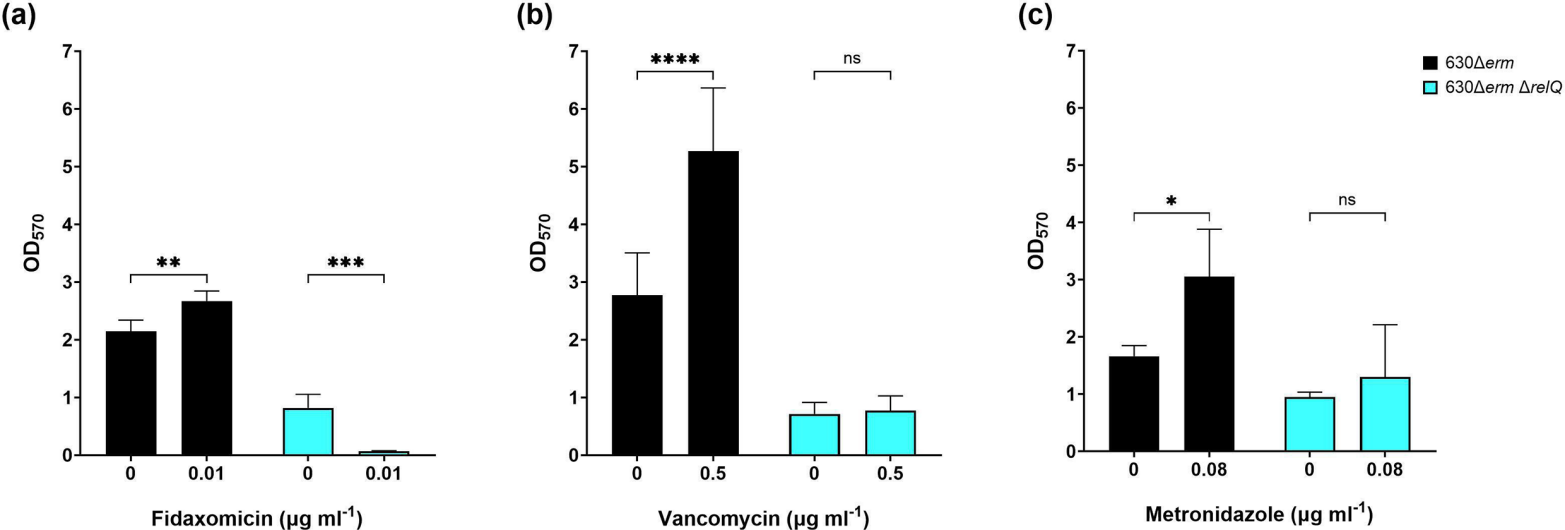
Effect of sublethal antibiotics on biofilm production by *C. difficile* 630Δ*erm* and 630Δ*erm* Δ*relQ* strains. Biofilms for both strains were grown for 24 h in the presence and absence of (**a**) 0.01 µg ml^−1^ fidaxomicin, (**b**) 0.5 µg ml^−1^ vancomycin or (**c**) 0.08 µg ml^−1^ metronidazole before washing and staining with crystal violet. Data represent the mean and standard deviation of at least four independent biologicals measured in duplicate. Conditions with antibiotics were compared to untreated controls by one-way ANOVA. ****P<0.0001; ***P<0.0005; **, *P*<0.01; *P<0.05; ns, not significant.

We initially tested biofilm formation in BHIS media containing 0.01 µg ml^−1^ fidaxomicin, 1.0 µg ml^−1^ vancomycin and 0.3 µg ml^−1^ metronidazole. These were the highest antibiotic concentrations that allowed for the growth of the wild-type strain (Fig. 2). The concentration of 1.0 µg ml^−1^ vancomycin did not show any noticeable effect on biofilm formation for both strains (data not shown). On the contrary, the presence of 0.3 µg ml^−1^ metronidazole did not stimulate biofilm formation for the wild-type strain but inhibited biofilm production for the deletion strain (data not shown). We subsequently tested sub-inhibitory concentrations of 0.01 µg ml^−1^ fidaxomicin, 0.5 µg ml^−1^ vancomycin and 0.08 µg ml^−1^ metronidazole and found that all these conditions stimulated a significant increase in 24 h biofilm formation by wild-type strain (Fig. 5). Contrary to the wild-type strain results, when the deletion strain was exposed to fidaxomicin, a significant decrease in biofilm formation was observed (Fig. 5a). Biofilm formation by the deletion strain showed no response to vancomycin or metronidazole stress in contrast to the increases observed by the wild-type strain (Fig. 5b, c).

**Fig. 6.**
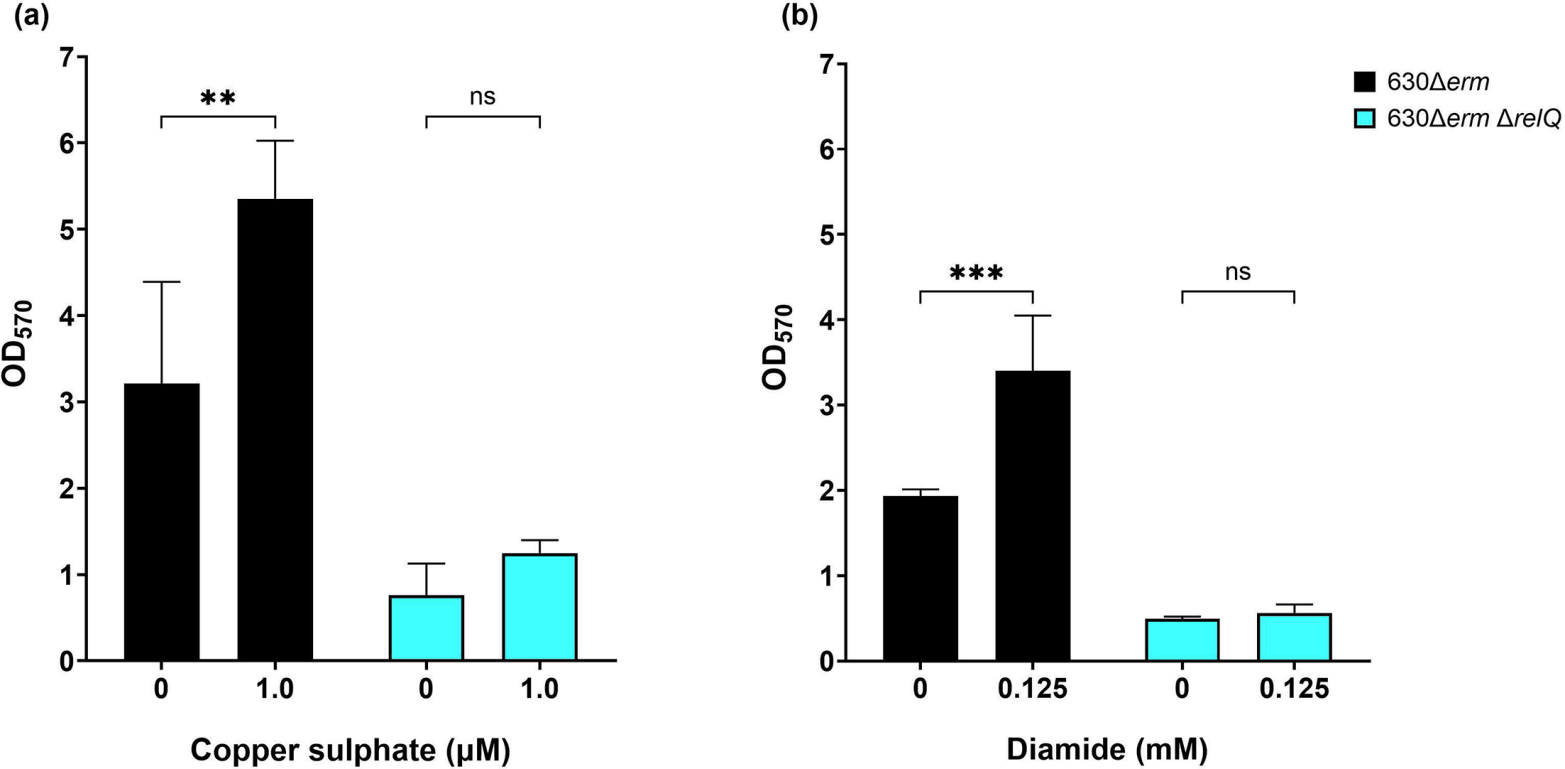
Effect of sublethal oxidants on biofilm production by *C. difficile* 630Δ*erm* and 630Δ*erm* Δ*relQ* strains. Biofilms were grown for 24 h in the presence and absence of (**a**) 1.0 µM copper sulphate and (**b**) 0.125 mM diamide. Data represent the mean and standard deviation of at least four independent biologicals measured in duplicate. Conditions with antibiotics were compared to untreated controls by one-way ANOVA. ***P<0.0005; **P<0.01; ns, not significant.

Similar results were observed for biofilm formation when both strains were subjected to sublethal oxidative stress (Fig. 6). Treatment with 2.0 µM copper sulphate had no discernible impact on biofilm production for the wild-type and deletion strains (data not shown). Decreasing the copper sulphate concentration to 1.0 µM resulted in a significantly higher amount of biofilm formation by the wild-type strain, while the deletion strain had no significant biofilm response to the copper stress (Fig. 6a). Treatment with 0.125 mM diamide had no effect on the growth of either strain (Fig. 3c, d). This sublethal diamide concentration stimulated a significant amount of biofilm formation by the wild-type strain, while the deletion strain was non-responsive (Fig. 6b).

### *relQ* deletion affects *C. difficile* spore accumulation in adherent biofilms and biofilm supernatants

**Fig. 7.**
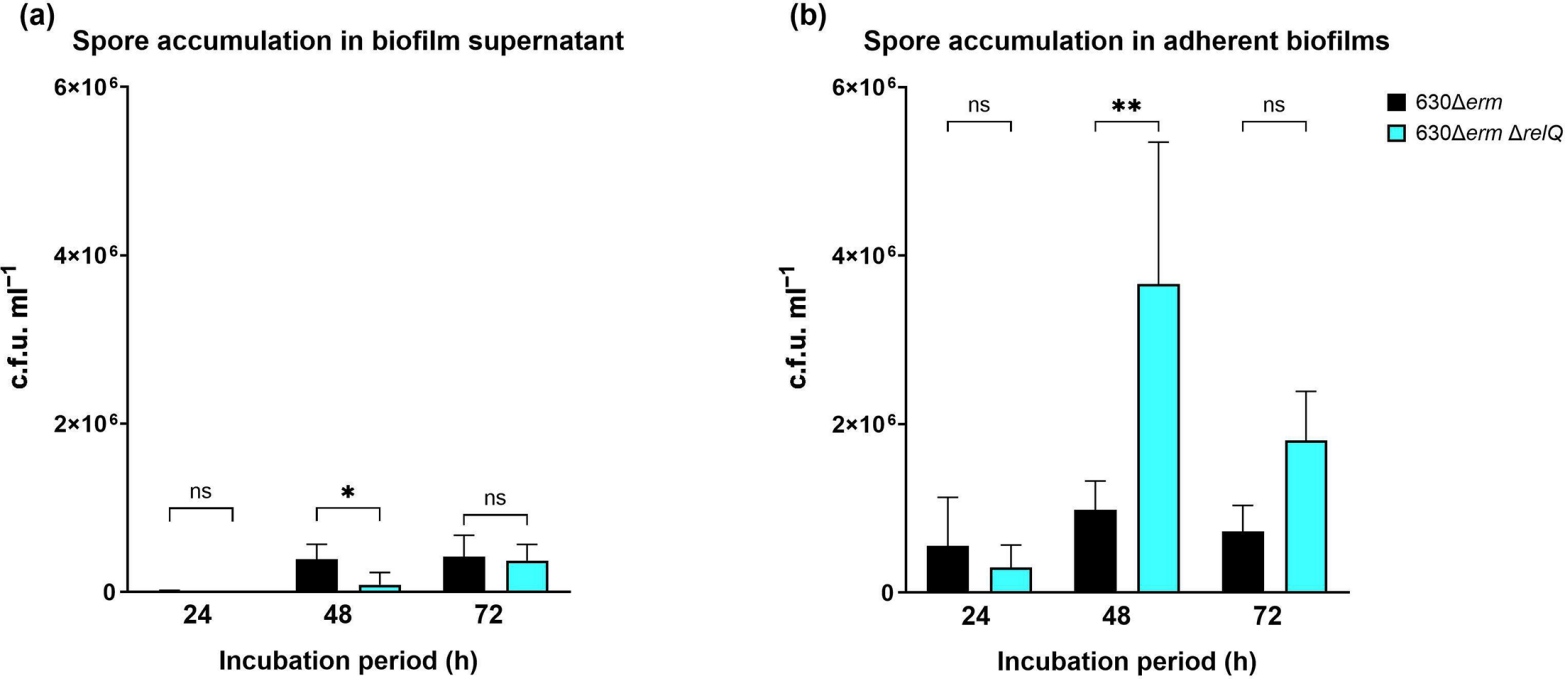
Spore accumulation in adherent biofilms and biofilm supernatant of *C. difficile* 630Δ*erm* and 630Δ*erm* Δ*relQ* strains. Spores of 630Δ*erm* and 630Δ*erm* Δ*relQ* were purified from (**a**) biofilm supernatant or (**b**) washed biofilms after 24, 48 or 72 h of incubation. Data represent the mean and standard deviation of at least three independent biologicals measured in duplicate. Measurements were compared between the strains by one-way ANOVA. **P<0.01; *P<0.05; ns, not significant.

Sporulation and biofilm formation contribute to *C. difficile* virulence and recurrent infections. However, their coordinated regulation is poorly understood. We wanted to examine accumulation of spores in adherent biofilms and their corresponding liquid-phase supernatants. After 24 h, the wild-type strain forms very few spores in biofilm supernatants (Fig. 7a). Between 24 and 48 h, an increase in the number of spores is observed in biofilm supernatant (Fig. 7a). Wild-type spore accumulation in biofilm supernatant does not increase substantially between 48 and 72 h (Fig. 7a). In the liquid supernatants separated from adherent biofilms, the deletion strain produces very few spores at 24 or 48 h. However, an increase in the number of viable deletion strain spores is observed between 48 and 72 h (Fig. 7a). In fact, the spore counts in supernatants after 72 h are similar between the two strains (Fig. 7a).

When the viable spores in washed biofilms were enumerated after the biofilms were washed, detached and treated with ethanol to kill vegetative cells, there was no significant difference between the wild-type and deletion strains after 24 h, but the deletion strain showed significantly more spores in the 48 h biofilms (Fig. 7b). The spore concentration in the wild-type biofilms showed no significant difference between 48 and 72 h. In contrast, deletion biofilms exhibited reduced spore counts after 72 h (Fig. 7b).

**Fig. 8.**
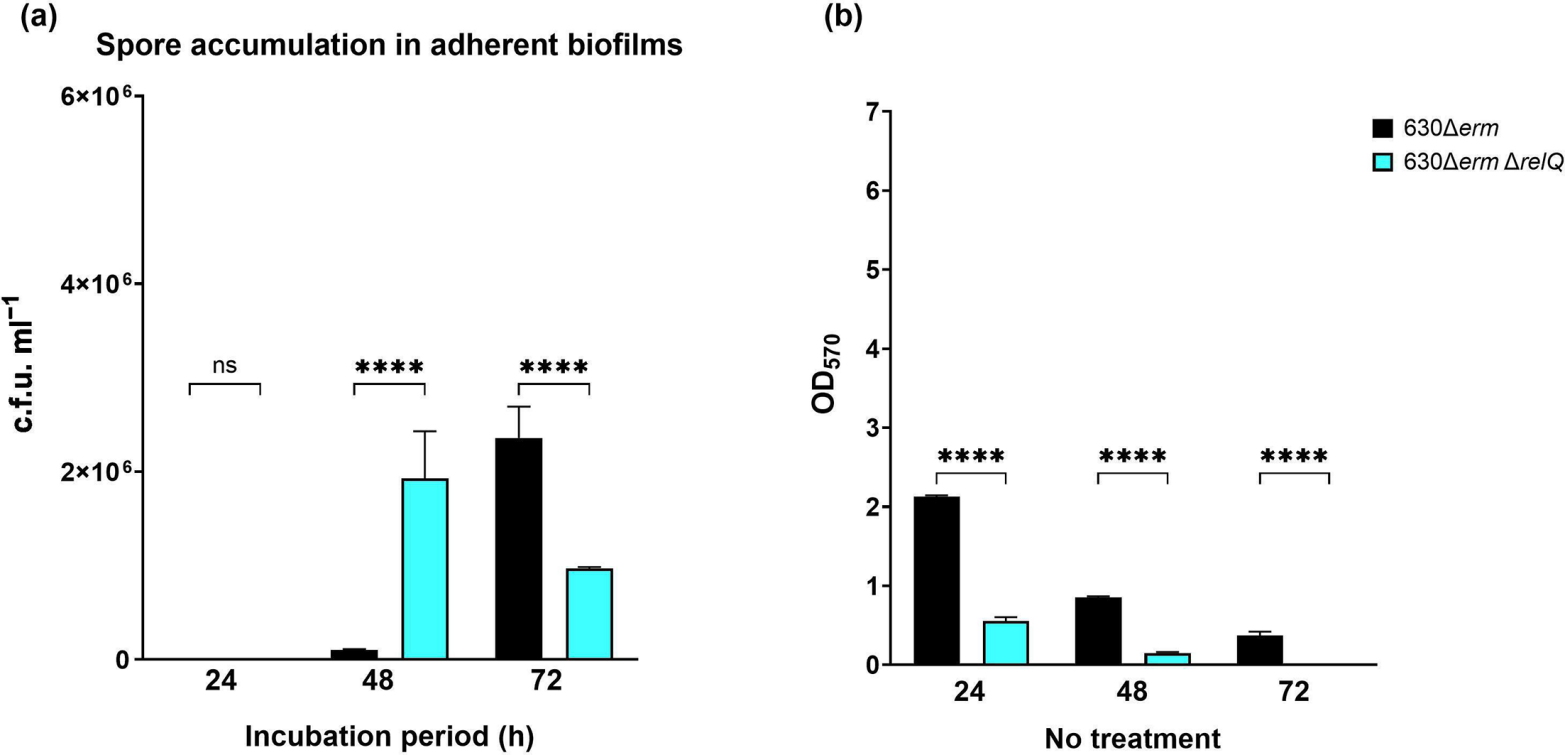
Spore accumulation in adherent biofilms and biofilm production by *C. difficile* 630Δ*erm* and 630Δ*erm* Δ*relQ* strains. (**a**) Spores of wild-type and deletion strains were purified from washed biofilms after 24, 48 or 72 h of incubation. (**b**) Biofilms were grown for 16, 24 or 48 h before washing and staining with crystal violet. Data represent the mean and standard deviation of at least three independent biologicals measured in duplicate. Measurements were compared between the strains by one-way ANOVA. ****P<0.0001; ns, not significant.

As both strains exhibited a reduction in biofilm biomass between 24 and 48 h (Fig. 4), we speculated that the drop in biofilm-bound deletion strain spores between 48 and 72 h could be due to a further decrease in adherent biofilm biomass. To assess this, we repeated our enumeration of biofilm-bound spores at 24, 48 and 72 h and measured the total biofilm biomass at each timepoint. While the number of spores enumerated varied between experiments, the progression of each strain over time was consistent. The deletion strain biofilms had accumulated significantly more spores than the wild-type strain biofilms at 48 h, despite having a much lower biofilm biomass. By 72 h, the number of spores in the wild-type biofilms had increased while that in the deletion strain biofilms had decreased (Fig. 8a). Both strains showed decreased biomass between 48 and 72 h (Fig. 8b). The decrease was more pronounced in the deletion strain, which had very little adherent biomass by 72 h (Fig. 8b).

### The effects of *relQ* deletion on biofilm can be complemented *in trans*

To verify that the observed phenotypes were due only to the absence of *relQ*, we complemented the deletion strain with *relQ* under the control of its native promoter on the plasmid pMC123 [62]. As the complemented strain was grown in the presence of thiamphenicol to maintain the plasmid, we also mated pMC123 into the deletion strain.

**Fig. 9.**
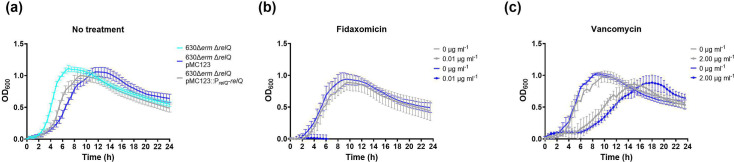
Growth of 630Δ*erm* Δ*relQ*, 630Δ*erm* Δ*relQ* pMC123 and 630Δ*erm* Δ*relQ* pMC123::P*_relQ_-relQ* strain cultures in (**a**) BHIS broth, (**b**) 0.01 µg ml^−1^ fidaxomicin and (**c**) 2.0 µg ml^−1^ vancomycin. Data represent the mean and standard deviation of three independent biologicals measured as duplicates.

Both the complemented strain and the deletion strain containing the control plasmid exhibit growth similar to that of the deletion strain in BHIS medium containing thiamphenicol but no other stressors, reaching maximum optical densities of 1.111, 0.998 and 1.057, for the knockout strain, complemented strain and deletion strain containing the control plasmid, respectively, before declining (Fig. 9a). Both strains exhibit slightly slower growth than that of the deletion strain, presumably due to the presence of thiamphenicol. Neither plasmid-bearing strain exhibits any growth in the presence of 0.01 µg ml^−1^ fidaxomicin (Fig. 9b), and 2.0 µg ml^−1^ vancomycin delays growth in both strains (Fig. 9c).

**Fig. 10.**
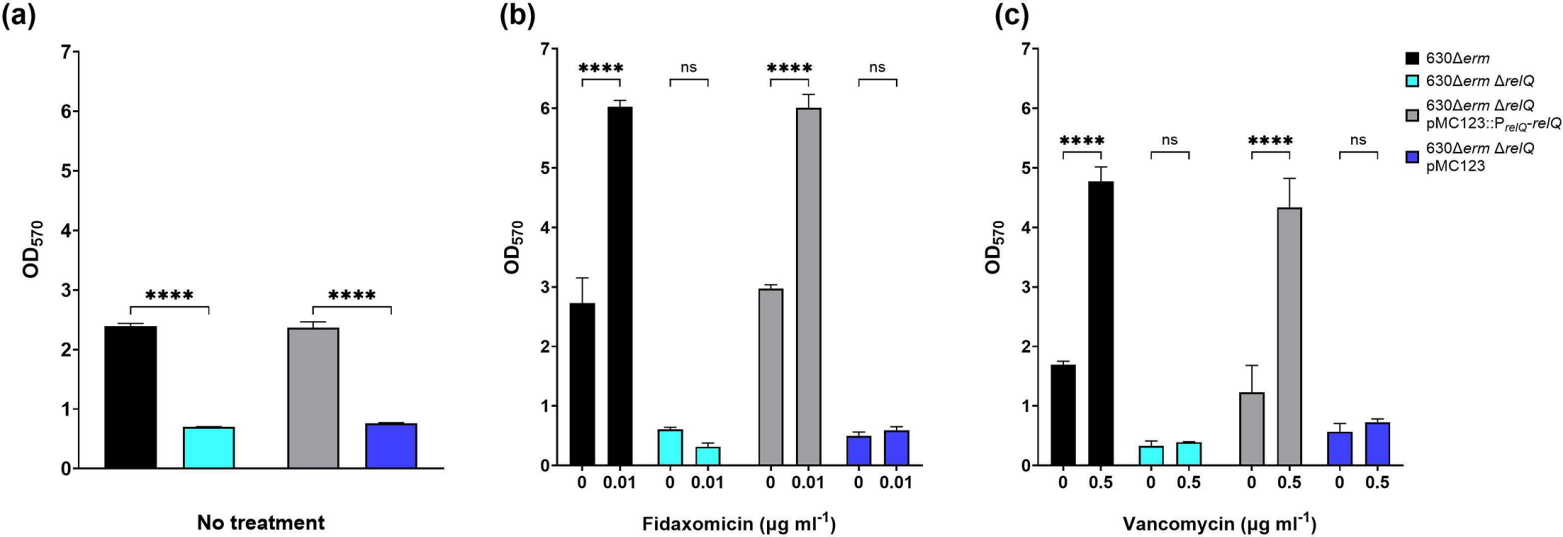
Effect of sublethal antibiotics on biofilm production by *C. difficile* 630Δ*erm*, 630Δ*erm* Δ*relQ*, 630Δ*erm* Δ*relQ* pMC123::P*_relQ_-relQ* and 630Δ*erm* Δ*relQ* pMC123 strains. (**a**) Biofilms were grown for 24 h in BHIS media and in the presence and absence of (**b**) 0.01 µg ml^−1^ fidaxomicin and (**c**) 0.5 µg ml^−1^ vancomycin. Biofilms were grown for 24 h before washing and staining with crystal violet. Data represent the mean and standard deviation of three independent biologicals measured in duplicate. Wild-type and deletion strain measurements at each timepoint and conditions with antibiotics were compared to untreated controls by one-way ANOVA. ****P<0.0001; ns, not significant.

The effects of complementation on biofilm formation were more pronounced. Biofilms formed by the complemented strain in BHIS containing thiamphenicol were indistinguishable from those formed by wild-type 630Δ*erm* in BHIS. Similarly, biofilms formed by the deletion strain carrying the vector control showed no significant difference from those formed by the deletion strain (Fig. 10a). In addition to enabling the formation of more robust biofilms, complementation also restored the deletion strain’s ability to increase biofilm formation after exposure to sublethal fidaxomicin and vancomycin stress. The deletion strain carrying the control vector did not increase biofilm formation when exposed to either antibiotic (Fig. 10b, c).

## Discussion

The classical starvation-induced SR in Gram-positive Firmicutes is understood to be primarily mediated by a single RSH/Rel family enzyme, sometimes named Rel or RelA, while RelP and RelQ small alarmone synthetases more commonly play roles in basal alarmone synthesis and responses to cell wall stress [63,71]. SASs have been linked to stress-induced biofilm formation in *S. aureus* [54]. The drop in cytoplasmic GTP levels that results from alarmone synthesis has been linked to sporulation in *B. subtilis*, but to our knowledge, alarmone-regulated sporulation in Firmicutes has not otherwise been demonstrated [58,72].

Our results reveal that *relQ* deletion has meaningful and stress-specific effects on the exponential growth, biofilm formation and spore accumulation of *C. difficile* 630Δ*erm* even in the presence of the intact bifunctional RSH/Rel. The absence of *relQ* disrupts the regulation of *C. difficile* 630Δ*erm* growth in unstressed conditions. However, the consequence of *relQ* deletion exhibits variability when the bacterium is exposed to sublethal stresses that were tested in this study. Without *relQ*, *C. difficile* 630Δ*erm* displays altered susceptibility to fidaxomicin and vancomycin but not to metronidazole or oxidants, underscoring the specificity of the RelQ-mediated response to individual extracellular stressors.

The observed growth phenotypes of the *relQ* deletion strain did not complement when the gene and promoter were restored *in trans*, although these assays were likely complicated by the presence of thiamphenicol to maintain the vector. Exposure to a sublethal stress has been documented to increase bacterial resilience against other stresses [73], so it is likely that employing plasmid-based thiamphenicol resistance altered the regulatory and metabolic landscape within the cells enough to affect RelQ-mediated growth processes. Because sublethal stresses can also affect sporulation [74], we did not assess the effect of complementation on spore accumulation within biofilms.

We have confirmed previous findings that exposure to sub-inhibitory antibiotic concentrations stimulated increased biofilm formation in wild-type *C. difficile* 630Δ*erm* and found that intact *relQ* is necessary for stress-induced biofilm upregulation. Complementation did restore biofilm formation and antibiotic-induced biofilm upregulation in the *relQ* deletion strain, suggesting that the unknown mechanisms by which RelQ affects biofilm formation are not affected by thiamphenicol stress. We also demonstrated that *C. difficile* increases biofilm accumulation in response to oxidative stress, which, to our knowledge, is the first report of this phenomenon and found that *relQ* is also necessary for oxidative stress-induced biofilm formation. As oxidative stress is employed by the innate immune system, this *relQ-*dependent biofilm stress response is likely to contribute to the high resilience of the pathogen within mammalian hosts [75].

*C. difficile* biofilms have a high affinity for spores *in vitro* and have been proposed to serve as a reservoir for spores *in vivo*, suggesting that biofilm production and sporulation are complementary processes that are coordinately regulated [24]. Deletion of the transcriptional regulator Spo0A abolishes sporulation in *C. difficile* R20291 and also results in notably diminished biofilm formation compared to the wild-type [21,76]. Biofilms formed by the *spo0A* mutant were prone to easy detachment from their growth surfaces and exhibited decreased resistance to oxygen stress [21]. In our study, the *ΔrelQ* strain exhibited less biofilm formation, lost the ability to upregulate biofilm in response to stress and altered the kinetics of spore accumulation in two different environments. The deletion strain accumulated spores more slowly in liquid broth and more rapidly in biofilms, despite having a lower total biofilm biomass than the wild-type at all timepoints. The loss of *relQ* resulted in a sparser biofilm containing a lower ratio of vegetative cells to associated spores. This spore-enriched biofilm is less persistent over the course of 72 h than that formed by the wild-type cells, which could lead to altered persistence within a mammalian host. Future work with mixed-species biofilms and animal studies will be necessary to determine whether RelQ limits the pace of biofilm spore accumulation in more complex biofilms and whether the loss of this regulation is detrimental to survival and pathogenesis. It also must be noted that these studies only include the *C. difficile* 630Δ*erm* background, which forms less robust biofilms *in vitro* than more recent epidemic strains such as R20291 [77]. As transcriptional regulation of the *rsh* gene has previously been shown to differ between 630Δ*erm* and R20291 [14], the role of RelQ in regulating clostridial biofilm formation will have to be experimentally determined in multiple strains to fully understand its role.

This current work establishes a role for RelQ-mediated alarmone signalling in growth, biofilm formation and spore accumulation within biofilms in one strain of *C. difficile*. These are all complex processes affected by multiple regulatory pathways, and the mechanism(s) by which alarmones can affect each are yet to be determined. As alarmones can serve as both transcriptional regulators and allosteric enzyme regulators, transcriptomic analysis and screening for alarmone-binding protein effectors in multiple genetic backgrounds will be necessary before alarmone signalling in *C. difficile* can be accurately modelled [10]. It is likely that in these studies, we have observed evidence of two different roles played by RelQ in *C. difficile*. The inability of the deletion strain to upregulate biofilm formation in response to antibiotic or oxidant exposure indicates that RelQ contributes to the clostridial response to some extracellular stresses independently of RSH/Rel. However, in the absence of additional stress, the deletion strain exhibits accelerated exponential vegetative growth and reduced spore accumulation in liquid culture, suggesting that RelQ also restrains unstressed *C. difficile* growth and proliferation. Biofilms formed by the deletion strain are less substantial but contain more spores at earlier timepoints than those formed by the wild-type strain. Cell division and differentiation into either free-living planktonic cells, matrix-producing vegetative biofilm cells or spores are coordinately regulated, and it appears that pGpp produced by RelQ in response to growth conditions affects the distribution of the *C. difficile* population among these pathways. Future examination of these processes in a pGpp-null (Δ*rsh*Δ*relQ*) strain could provide even more insight into the non-redundant functions of RSH/Rel and RelQ in this organism. It is clear that alarmone signalling affects many aspects of * C. difficile* physiology with potential relevance to infection persistence and recurrence.

## Methods

### Bacterial strains and growth conditions

Table S1 lists all the plasmids and bacterial strains that were used in this study. Table S2 lists all the primers that were used in this study. *C. difficile* strains were grown at 37 °C in brain heart infusion (BHI; VWR) medium supplemented with 5.0 % yeast extract (VWR) [78]. These strains were cultivated in a Coy anaerobic chamber (Coy Laboratory) with an atmosphere of 10.0 % CO_2_, 5.0 % H_2_ and 85.0 % N_2_. *E. coli* strains were grown in Luria-Bertani (LB; Thermo Fisher Scientific) medium at 37 °C in a MaxQ 6000 incubator (Thermo Fisher Scientific). Bacterial strains that carried plasmids were maintained using the following antibiotics at the indicated concentrations: 4 or 50 µg ml^−1^ ampicillin (Amp) (Thomas Scientific), 100 ng ml^−1^ anhydrotetracycline (ATc) (Thomas Scientific), 15 µg ml^−1^ chloramphenicol (Cm) (Thomas Scientific), 10 µg ml^−1^ thiamphenicol (Tm) (Thermo Fisher Scientific) and 100 µg ml^−1^ kanamycin (Kan) (Thomas Scientific). All plastic consumables were brought into the anaerobic chamber for equilibration for a minimum of 72 h before use.

### Plasmid and strain construction

The pMSR vector (provided by Olga Soutourina), containing the toxin gene *CD2517.1* for counter-selection, was used for ACE in *C. difficile* 630Δ*erm,* as described in reference [79]. It was maintained in *E. coli* DH5α cells (EP109 in Table S1). NEBuilder HiFi DNA assembly was used to amplify the ‘RelQ arms’, the upstream and downstream homology regions flanking *C. difficile* 630Δ*erm relQ* (CD630DERM_03450). The RelQ arms were amplified using primer pairs RelQ Arm 1_F+Arm 1_R and Arm 2_F+Arm 2_R (Table S2). The vector and amplified arms were digested using the PmeI cut site (NEB). The arms and vector were ligated using the Gibson Assembly Cloning Kit (NEB), thus generating pMSR::P*_relQ_-relQ*, which was transformed into competent *E. coli* DH5α cells (EP111 in Table S1). The plasmid was confirmed by PCR using primer pair, RelQ Arm 1_F and Arm 2_R (Table S2). The pMSR::P*_relQ_-relQ* plasmid was then transformed into competent *E. coli* HB101 cells carrying the pRK24 plasmid (EP113 in Table S1) to create the donor strain for conjugative transfer. The pRK24 plasmid is a derivative of a broad-host-range plasmid RP4, which is involved in the transfer of plasmid DNA into *C. difficile* strains [80].

The pMC123 plasmid (provided by Shonna M. McBride) was sustained in *E. coli* DH5α cells (RT264 in Table S1). The pMC123 plasmid was subsequently transformed into competent *E. coli* HB101 cells carrying the pRK24 plasmid to create the empty vector donor strain (EP134 in Table S1) for conjugative transfer into *C. difficile* 630Δ*erm* Δ*relQ*. To create the complement strain, NEBuilder HiFi DNA assembly was utilized to amplify the ‘RelQ arms’, which are the upstream and downstream homology regions flanking the *relQ* gene in *C. difficile* 630Δ*erm* (CD630DERM_03450). Primer pairs RelQ Arm 1_F+Arm 1_R and Arm 2_F+and Arm 2_R (Table S2) were used to amplify the RelQ arms. The pMC123 plasmid and amplified arms were digested at the EcoRI and PstI cut sites (NEB). The arms and plasmid were ligated using a 1 : 3 (plasmid/arms) ratio using T4 DNA ligase (NEB), creating pMC123::P*_relQ_-relQ* plasmid, which was then transformed into competent *E. coli* DH5α cells (EP131 in Table S1). The plasmid was confirmed by PCR using primer pair RelQ Arm 1_F and Arm 2_R (Table S2). The pMC123::P*_relQ_-relQ* plasmid was subsequently transformed into competent *E. coli* HB101 cells harbouring the pRK24 plasmid (RT270 in Table S1) to create the complement donor strain for conjugative transfer (EP133 in Table S1).

To mate plasmids into *C. difficile*, a single colony of the donor strain was inoculated in 3 ml of LB media supplemented with 15 µg ml^−1^ Cm and 50 µg ml^−1^ Amp. The culture was incubated for 14–16 h at 37 °C with continuous shaking at 250 r.p.m. The overnight culture was plated on LB agar plates supplemented with 15 µg ml^−1^ Cm and 50 µg ml^−1^ Amp. The plates were incubated overnight at 37 °C. Inside the anaerobic chamber, a single colony of the acceptor strain was inoculated in 3 ml of BHIS broth, which was then incubated at 37 °C for 14–16 h. The lawns of *E. coli* donor cells were scraped from the agar plate and resuspended in 1 ml LB broth, centrifuged and resuspended in 150 µl LB broth. Inside the anaerobic chamber, a 1 : 1 ratio of both donor and acceptor cell suspensions was mixed, and 50 µl spots were pipetted onto BHIS agar plates supplemented with 11 mM glucose (Thermo Fisher Scientific) and 4 µg ml^−1^ Amp. The plates were incubated at 37 °C for 18 h inside the anaerobic chamber. After the 18 h incubation, the spots were scraped up with a sterile inoculating loop and suspended in 1 ml of BHIS broth before 100 µl of suspension was spread onto BHIS agar plates supplemented with 10 µg ml^−1^ Tm and 100 µg ml^−1^ Kan. These selective plates were incubated at 37 °C for 20–24 h. Colonies were then streaked twice onto fresh BHIS agar plates supplemented with 10 µg ml^−1^ Tm and 100 µg ml^−1^ Kan. Strains carrying pMC123 or pMC123::P*_relQ_-relQ* were passaged on BHIS agar plates containing 10 µg ml^−1^ Tm and confirmed using PCR with primer pair RelQ Arm 1_F and Arm 2_R (Table S2). Strains carrying pMSR::P*_relQ_-relQ* were passaged onto BHIS agar plates supplemented with 10 µg ml^−1^ Tm to select for plasmid integration into the chromosome. The resulting colonies were streaked twice on BHIS agar plates supplemented with 100 ng ml^−1^ ATc to induce expression of the plasmid-encoded *CD2517.1* toxin and plasmid excision. These counter-selected colonies were then simultaneously streaked onto BHIS agar plates with and without 10 µg ml^−1^ Tm, and only colonies that had regained sensitivity to thiamphenicol were screened as potential deletion strains. Potential deletion strains were confirmed by PCR with primer pair RelQ Arm1_F and RelQ Arm 2_R (Table S2, Fig. S1).

### Growth curves

Cultures were prepared by inoculating individual colonies into 3 ml of BHIS broth with or without 10 µg ml^−1^ Tm as indicated and grown for 14–16 h at 37 °C in the anaerobic chamber. The overnight cultures were diluted 1 : 20 into BHIS broth containing the indicated concentrations of thiamphenicol (Alfa Aesar), fidaxomicin (Cayman Chemical), vancomycin (VWR), metronidazole (VWR), copper sulphate (Fisher Scientific) or diamide (MP Biomedicals) into sterile 96-well plates (Fisher Scientific). The plates were incubated at 37 °C for 24 h in a Stratus microplate reader (Stellar Scientific), which was set to record the optical density at 600 nm every 30 min.

### Biofilm formation, visualization and quantification

Overnight cultures were diluted 1 : 10 into BHIS broth containing the indicated concentrations of thiamphenicol, fidaxomicin, vancomycin, metronidazole, copper sulphate or diamide. Biofilms were grown in a total volume of 2 ml in 24-well sterile non-treated plates (Corning) under anaerobic conditions at 37 °C for the indicated times. After incubation, the plates were removed from the chamber and the exteriors were sterilized with SporGon (Thomas Scientific), 10 % bleach and 70 % ethanol (VWR). The supernatant was removed by pipetting, and the adherent biofilms were washed with 1 ml of 1× PBS solution. The washed biofilms were stained with 1 ml of 0.1 % crystal violet (Sigma-Aldrich) in each well for 30 min at room temperature. After the 30 min incubation, the crystal violet was removed, and the stained biofilms were washed twice with 1 ml of 1× PBS. Stained biofilms were scraped up with sterile pipette tips and suspended in 1 ml of 70 % ethanol. A Bio-Tek synergy plate reader (Marshall Scientific) was used to record the optical density at 570 nm after shaking the plate at medium intensity.

### Viability assay of spores in biofilm supernatants

Individual colonies of *C. difficile* 630Δ*erm* and 630Δ*erm* Δ*relQ* strains were inoculated into 3 ml of BHIS broth. The cultures were incubated for 24, 48 or 72 h at 37 °C in a Coy anaerobic chamber. After the indicated incubation, duplicate 470 µl aliquots of each culture were removed from the chamber in 2 ml Eppendorf tubes (VWR), which were sterilized with SporGon, 10 % bleach and 70 % ethanol. The experimental tubes were opened and exposed to oxygen for 1 h and treated with 530 µl of 95 % ethanol, to get a final concentration of 50 % ethanol, for 1 h with periodic mixing every 15 min, while the controls remained closed and were not treated with ethanol. After the 1 h treatment, the samples were brought back into the chamber and subsequently underwent serial dilution in BHIS broth. Aliquots of 100 µl from the original and diluted samples were plated as duplicates on BHIS agar with and without 0.1 % taurocholic acid. The plates were incubated at 37 °C for 48 h before the viable colonies were counted. The range for viable colony count was restricted between 20 and 350.

### Viability assays of spores in adherent biofilms

Biofilms were inoculated, as described in the ‘Biofilm formation, visualization, and quantification’ methodology section, and incubated anaerobically at 37 °C for 24, 48 or 72 h. After the respective incubation, the plates were brought out of the chamber and the exteriors were sterilized. The supernatant was discarded, and the adhered biofilms in each well were washed with 1 ml of 1× PBS. The biofilms were allowed to dry before being washed again with 1 ml of 1× PBS. The washed biofilms were scraped up in each well with a sterile pipette tip before being suspended in 1 ml of BHIS broth. The samples were taken into the chamber where they underwent a serial dilution in BHIS broth before being plated as duplicates on BHIS agar with and without 0.1 % taurocholic acid. The plates were incubated at 37 °C for 48 h before the viable colonies were counted. The range for viable colony count was restricted between 20 and 300.

### Statistical analyses

The growth curve, biofilm and spore accumulation data are presented as mean±standard deviation for each experiment. Prism 10 (GraphPad) was used for all statistical calculations, data fitting and plotting. Quantitative experiments were performed as duplicates and were repeated at least three times.

## supplementary material

10.1099/mic.0.001479 Uncited Supplementary Material 1.

## Abbreviations

ACE: allele-coupled exchange
Amp: ampicillin
aTc: anhydrotetracycline
BHIS: brain-heart infusion supplemented
CDI: *C. difficile* infection
Cm: chloramphenicol
GDP: guanosine-5'-diphosphate
GMP: guanosine-5'-monophosphate
GTP: guanosine-5'-triphosphate
Kan: kanamycin
LB: Luria-Bertani
OD: optical density
(pp)pGpp: nucleotide signaling alarmones pppGpp, ppGpp, and/or pGpp
RelQ: clostridial small alarmone synthetase
RSH: RelA/SpoT homology family
SAH: small alarmone hydrolase
SAS: small alarmone synthetase
SR: stringent response
Tm: thiamphenicol
