# Supplementary material for: RelQ-mediated alarmone signalling regulates growth, stress-induced biofilm formation and spore accumulation in Clostridioides difficile
Source: Microbiology (Reading). 2024 Jul 19;170(7):001479. doi: 10.1099/mic.0.001479 (PMC11317968; doi:10.1099/mic.0.001479)
Supplement: Uncited Supplementary Material 1. [file mic-170-01479-s001.pdf]

Table S1 Strains and plasmids used in this paper.

**Plasmids**		
**Name**	**Description**	**Reference**
pMSR	ACE vector for chromosomal manipulation of *C. difficile* 630 using a counter-selection marker (CD2517.1 toxin)	[1]
pRK24	Used to facilitate in the transfer of plasmid DNA into *C. difficile* strains.	[2]
pMSR::P_{relQ}-*relQ*	Modified pMSR ACE vector with 630Δ*erm relQ* flanking sequences to target the *relQ* gene	This study
pMC123	Shuttle vector for *E. coli* to *C. difficile* conjugation	[3]
pMC123::P_{relQ}-*relQ*	Modified pMC123 vector with 630Δ*erm relQ* flanking sequences to target the *relQ* gene	This study
Escherichia coli		
**Name**	**Description**	
DH5α	Gentoype: *fhuA2Δ(argF-lacZ)U169 phoA glnV44 Φ80Δ(lacZ)M15 gyrA96 recA1 relA1 endA1 thi-1 hsdR17*	NEB
EP109	pMSR in DH5α	This study
EP111	pMSR::P_{relQ}-*relQ* in DH5α	This study
RT270	pRK24 in HB101	This study
EP113	pMSR::P_{relQ}-*relQ* and pRK24 in HB101	This study
RT264	pMC123 in DH5α	This study
EP131	pMC123::P_{relQ}-*relQ* in DH5α	This study
EP133	pMC123::P_{relQ}-*relQ* and pRK24 in HB101	This study
EP134	pMC123 and pRK24 in HB101	This study
Clostridioides difficile		
**Name**	**Description**	
630Δ*erm*	CD630 strain lacking *erm*B	[4]
CEP46	630Δ*erm* Δ*relQ*	This study
CEP50	630Δ*erm* Δ*relQ* pMC123::P_{relQ}-*relQ*	This study
CEP51	630Δ*erm* Δ*relQ* pMC123	This study

Table S2. Oligonucleotide primer sequences used in this paper.

Cloning primers		
**Name**	**Description and Sequence (5'-3')**	**Reference**
EP143	RelQ Arm 1_fwd<br>TTTTTTGTTACCCTAAGTTTGTTCAAGACCTAGGAGAG	This study
EP144	RelQ Arm 1_rev<br>GCCCCATTAAGCTCACCCTTTATTTGTTTTTTATG	This study
EP145	RelQ Arm 2_fwd<br>AAGGGTGAGCTTAATGGGGCTGCCTCTTAATG	This study
EP146	RelQ Arm 2_rev<br>AGATTATCAAAAAGGAGTTTCCGAAATCCCTCAAATATTTATTTTTAATC	This study

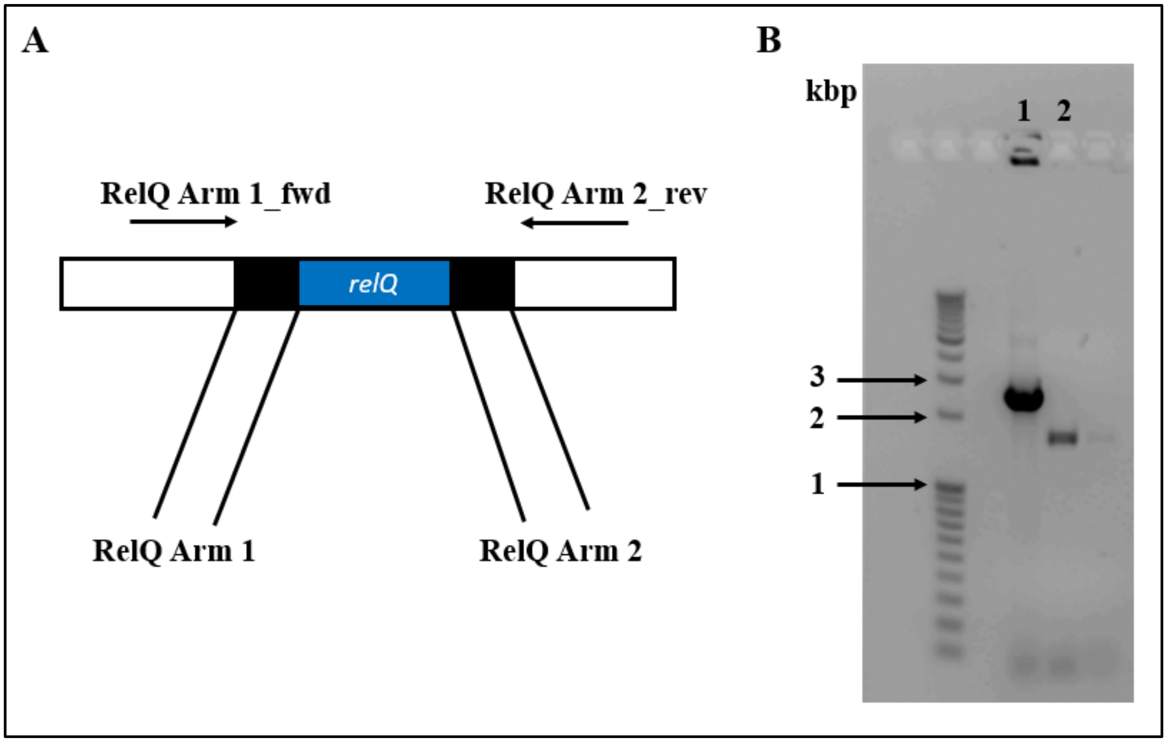

FIG S1 Confirmation of presence and absence of *relQ* in *C. difficile* 630Δ*erm* and *C. difficile* 630Δ*erm* Δ*relQ* strains. (A) Pictured are the *relQ* open reading frame (blue) and the amplified flanking regions (black). The primers RelQ Arm1_fwd and RelQ Arm2_rev amplify a 2.5 kbp region from the wild-type genome. In the Δ*relQ* strain, only the flanking regions are present and these primers amplify a 1.7 kbp product. (B) The products amplified from *C. difficile* 630Δ*erm* (lane 1) and *C. difficile* 630Δ*erm* Δ*relQ* (lane 2) using RelQ Arm1_fwd and RelQ Arm2_rev are pictured on a 0.8% DNA agarose gel.

REFERENCES

1. Peltier, J., et al., *Type I toxin-antitoxin systems contribute to the maintenance of mobile genetic elements in Clostridioides difficile.* Communications biology, 2020. **3**(1): p. 1-13.
2. Purcell, E.B., et al., *Cyclic diguanylate inversely regulates motility and aggregation in Clostridium difficile.* Journal of bacteriology, 2012. **194**(13): p. 3307-3316.
3. McBride, S.M. and A.L. Sonenshein, *Identification of a genetic locus responsible for antimicrobial peptide resistance in Clostridium difficile.* Infection and immunity, 2011. **79**(1): p. 167-176.
4. Hussain, H.A., A.P. Roberts, and P. Mullany, *Generation of an erythromycin-sensitive derivative of Clostridium difficile strain 630 (630Δ erm) and demonstration that the conjugative transposon Tn 916 ΔE enters the genome of this strain at multiple sites.* Journal of medical microbiology, 2005. **54**(2): p. 137-141.
